# Decreased Circulating T Regulatory Cells in Egyptian Patients with Nonsegmental Vitiligo: Correlation with Disease Activity

**DOI:** 10.1155/2015/145409

**Published:** 2015-12-15

**Authors:** Doaa Salah Hegab, Mohamed Attia Saad Attia

**Affiliations:** ^1^Faculty of Medicine, Dermatology and Venereology Department, Tanta University Hospitals, El Geish Street, Tanta 31111, Egypt; ^2^Faculty of Medicine, Clinical Pathology Department, Tanta University Hospitals, El Geish Street, Tanta 31111, Egypt

## Abstract

*Background*. Vitiligo is an acquired depigmentary skin disorder resulting from autoimmune destruction of melanocytes. Regulatory T cells (Tregs), specifically CD4^+^CD25^+^ and Forkhead box P3^+^ (FoxP3^+^) Tregs, acquired notable attention because of their role in a variety of autoimmune pathologies. Dysregulation of Tregs may be one of the factors that can break tolerance to melanocyte self-antigens and contribute to vitiligo pathogenesis.* Methods*. In order to sustain the role of Tregs in pathogenesis and disease activity of vitiligo, surface markers for CD4^+^CD25^+^ and FoxP3^+^ peripheral Tregs were evaluated by flow cytometry in 80 Egyptian patients with nonsegmental vitiligo in addition to 60 healthy control subjects and correlated with clinical findings.* Results*. Vitiligo patients had significantly decreased numbers of both peripheral CD4^+^CD25^+^ and FoxP3^+^ T cells compared to control subjects (11.49%  ± 8.58% of CD4^+^ T cells versus 21.20%  ± 3.08%, and 1.09%  ± 0.96% versus 1.44%  ± 0.24%, resp., *P* < 0.05 for both). Peripheral numbers of CD4^+^CD25^+^ and FoxP3^+^ Tregs correlated negatively with VIDA score.* Conclusion*. Treg depletion with impaired immune downregulatory function might play a key role in the autoimmune conditions beyond nonsegmental vitiligo particularly in active cases. Effective Treg cell-based immunotherapies might be a future hope for patients with progressive vitiligo.

## 1. Introduction

Vitiligo is an acquired cutaneous disorder characterized by progressive, selective destruction of melanocytes and clinically characterized by the development of milky white macules and patches [1]. It is a multifactorial polygenic disorder and several theories have been proposed about the pathogenesis of vitiligo including autoimmune hypothesis, reactive oxygen species model, zinc-*α*2-glycoprotein deficiency hypothesis, viral theory, intrinsic theory, and biochemical, molecular, and cellular alterations accounting for loss of functioning melanocytes [2]. The integration of epidemiological, clinical, histoimmunological, and therapeutic data strongly supports immunological pathomechanism in vitiligo, which is commonly associated with autoimmune diseases like autoimmune thyroid diseases, insulin-dependent diabetes mellitus, alopecia areata, and pernicious anemia [3–5].

T regulatory cells (Tregs) are a component of the immune system that constitutes a key mechanism in maintaining peripheral self-tolerance through the control of autoreactive lymphocyte activation and the prevention of harmful effects of such activation. These cells are involved in shutting down immune responses after they have successfully eliminated invading organisms [6]. Natural Tregs express the transcriptional factor Forkhead box P3 (FoxP3), which serves as the dedicated mediator of the genetic program for Tregs development and function. FoxP3^+^ is a reliable and specific marker of Tregs [7]. Natural and induced Tregs have many subsets with the most well-understood being those that express CD4, CD25, and FoxP3 (CD4^+^CD25^+^ FoxP3^+^ Tregs), which had acquired notable attention because of their role in a variety of autoimmune pathologies, inflammatory disorders such as asthma and colitis, and immune responses to tissue transplants, tumors, and various infectious agents pathologies [8]. There are several other subpopulations of Tregs such as Treg17 cells [9]; interleukin 10- (IL-10-) producing “Tr1” cells; transforming growth factor-*β*- (TGF-*β*-) producing T helper type 3 cells; CD8^+^ T suppressor cells; natural killer T cells; CD4^−^CD8^−^ T cells; and *γδ* T cells [10].

An altered generation of Tregs or a decrease in their suppressive functions may tip the balance towards autoimmunity, triggering the destruction of melanocytes and development of vitiligo [11].

This study aimed to evaluate the alterations in the numbers of peripheral CD4^+^CD25^+^ and FoxP3^+^ Treg lymphocytes by flow cytometry in a sample of Egyptian patients with nonsegmental vitiligo versus health controls and to correlate these alterations with clinical findings of cases including vitiligo disease severity and activity.

## 2. Materials and Methods

### 2.1. Study Groups

The present case-control study enrolled 80 Egyptian patients with nonsegmental vitiligo, including 36 males (45%) and 44 females (55%), in addition to 60 age- and sex-matched healthy volunteers as control subjects, including 36 males (60%) and 24 females (40%).  Table 1 shows the included subjects' demographic and clinical features. Vitiligo Area Scoring Index (VASI) [12] and Vitiligo Index of Disease Activity (VIDA) score [13] were determined for vitiligo patients. Patients who received systemic steroids or phototherapy for vitiligo in the preceding 6 weeks were excluded. Patients with comorbidities of other dermatologic or systemic diseases that may affect Tregs were also excluded. Two mL of peripheral venous blood was collected from all included subjects during their visits to the outpatient clinics of Dermatology and Venereology Department of Tanta University Hospitals after signing an informed written consent. Research approval was obtained from the Institutional Ethical Committee of Tanta University.

### 2.2. Antibodies Used and Flow Cytometric Analysis

The following monoclonal antibodies to human cell-surface molecules were used: anti-CD4-PerCP, anti-CD25-PE, and anti-FoxP3-APC, while the negative controls used were goat IgG-PerCP, IgG-PE, and IgG-APC (Becton-Dickinson Immunocytometry Systems, San Jose, CA, USA). Fluorocytometry was performed with a flow cytometer (FACSCalibur; Becton-Dickinson Immunocytometry Systems) equipped with a 488 nm blue laser (488-nm) and a red diode laser (635 nm) for multicolour fluorescence, plus forward-scatter and side-scatter measurements. Automated CellQuest Pro software (Becton-Dickinson Immunocytometry Systems) was used for fluorocytometric data analysis and graphic display, and the instrument was set by using calibrated beads provided. A minimum of 10.000 cells was measured in each analysis.

### 2.3. Statistical Analysis

Data were statistically analyzed by using Statistical Package for Social Sciences (SPSS Version 18). Data were expressed as the mean ± standard deviation, unless otherwise specified. Flow cytometric data were compared statistically among the groups by using the Mann-Whitney *U* test. The correlation coefficient (*r*) was generated by using Spearman's rank correlation. A *P* value of less than 0.05 was considered a statistically significant difference.

## 3. Results

### 3.1. Numbers of Peripheral CD4^+^CD25^+^ T Cells in Study Participants

**Figure 1:**
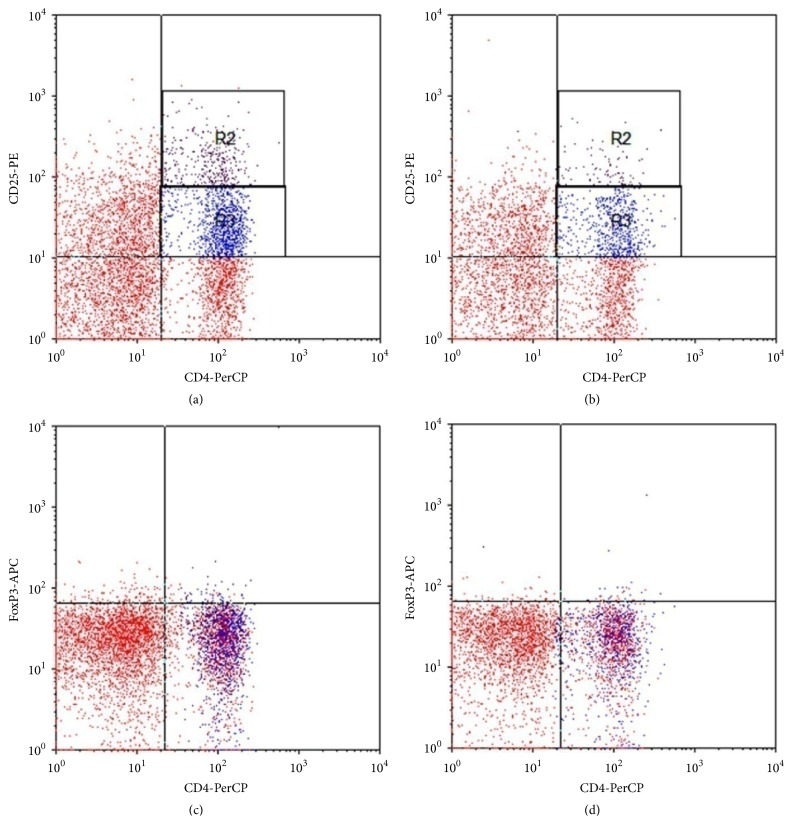
Frequency of circulating CD4^+^CD25^+^ Tregs and CD4^+^FoxP3^+^ T cells in healthy subjects and nonsegmental vitiligo patients by flow cytometry dot plot. (a) and (b) Representative profiles demonstrating Tregs as the CD4^+^CD25^high^ (R2) and CD4^+^CD25^low^ (R3) T-cell fraction by FACS analysis ((a) in a healthy subject, (b) in a vitiligo patient). (c) and (d) Representative profiles demonstrating Tregs as FoxP3^+^ CD4 T cells ((c) in a healthy subject, (d) in a vitiligo patient).

Treg cells were identified within peripheral CD4^+^ T-cell population according to their expression level of CD25 (Figures 1(a) and 1(b)). CD4^+^CD25^+^ T cells were CD4^+^ T-cell subsets with bright CD25 surface expression and were defined accordingly (including both CD4^+^CD25^low^ and, more importantly, CD4^+^CD25^high^).

**Figure 2:**
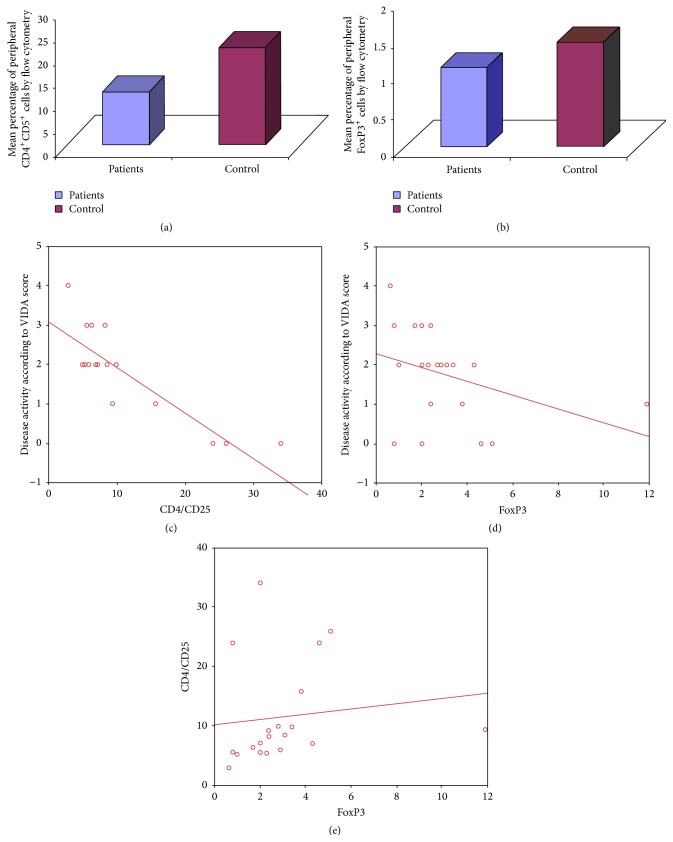
(a) The mean percentage of peripheral CD4^+^CD25^+^ Treg was significantly reduced in vitiligo compared to healthy controls. (b) The mean percentage of peripheral FoxP3^+^ T cells from total peripheral CD4^+^ cells was significantly reduced in vitiligo patients compared to controls. (c) Correlation between percentage of peripheral CD4^+^CD25^+^ lymphocytes and VIDA score. (d) Correlation between percentage of peripheral FoxP3^+^ lymphocytes and VIDA score. (e) Correlation between percentage of peripheral FoxP3^+^ lymphocytes in vitiligo patients group and the percentage of CD4^+^CD25^+^ lymphocytes in vitiligo patients.

Our results showed a statistically significant decrease in the percentage of peripheral CD4^+^CD25^+^ T cells in vitiligo patients (range from 2.9% to 34% of CD4^+^ T cells with a mean of 11.49%  ± 8.58%) compared to healthy controls (range from 17% to 27% with a mean of 21.20%  ± 3.08%) with *P* value <0.05 (Figure 2(a)).

### 3.2. Numbers of Peripheral FoxP3^+^ Tregs in Study Participants

FoxP3 expression was determined among the peripheral CD4^+^ T cells (Figures 1(c) and 1(d)). The percentage of peripheral FoxP3^+^ Tregs in vitiligo patients group ranged from 0.6% to 2.1% of CD4^+^ T cells with a mean of 1.09%  ±  0.96% which was significantly lower than that of control group which ranged from 0.04% to 2.0% with a mean of 1.44%  ± 0.24% (*P* < 0.05) (Figure 2(b)).

### 3.3. Correlation of the Numbers of Peripheral CD4^+^CD25^+^ T Cells and FoxP3^+^ Tregs with the Patients' Clinical Findings and with Each Other

A statistically significant negative correlation was observed between the percentage of CD4^+^CD25^+^ T cells and FoxP3^+^ Tregs in the peripheral blood of vitiligo patients and vitiligo disease activity according to VIDA score (*r* = −0.851, *P* < 0.001 and *r* = −0.512, *P* < 0.05, resp.) (Figures 2(c) and 2(d)), while a statistically significant positive correlation was observed between peripheral percentage of FoxP3^+^ Tregs and the percentage of CD4^+^CD25^+^ cells in vitiligo patients (*r* = 0.369, *P* < 0.05) (Figure 2(e)).

Each of circulating CD4^+^CD25^+^ Tregs percentage and FoxP3^+^ Tregs percentage did not correlate with patients' age, vitiligo disease duration, or VASI score.

## 4. Discussion

Immunological self-tolerance is maintained not only by deletion of self-reactive lymphocytes in the central lymphoid organs but also by the control of their activation and expansion in the periphery [14]. As a key mechanism of such peripheral self-tolerance, naturally occurring Tregs suppress the expansion/activation of self-reactive T cells which have escaped thymic negative selection. The majority of these Tregs constitutively express CD25^+^ (IL-2R *α*-chain) and constitute about 5–10% of peripheral CD4^+^ T cells in mice and humans [15]. FoxP3^+^ can be specifically expressed in CD4^+^CD25^+^ Treg cells and it is associated with their development and function [16].

The role of Tregs in vitiligo pathogenesis has been a recent topic of research, and there is evidence that Tregs are jeopardized in vitiligo patients [2]. However, there is some contradicting data and this subject demands further investigation.

In our study, the percentage of CD4^+^CD25^+^ T cells in the peripheral blood of vitiligo patients was significantly lower than healthy controls. This matches the results of several previous reports [17–19].

On the other hand, some other studies had reported a nonsignificant increase in the peripheral percentage of CD4^+^CD25^+^ T cells in vitiligo patients compared to healthy controls [20, 21]. In another report, the percentage of CD4^+^CD25^+^ T cells was significantly elevated in the vitiligo patient cohort relative to normal control donors and this finding had been considered as a protective mechanism against the autoimmune process. Nevertheless, their observation that peripheral blood levels of these cells apparently did not correlate with severity of the disease or its rate of progression suggested that they may be induced simply by occurrence of the disease and do not correlate directly with autoimmune processes [22].

In the current study a negative correlation was observed between the percentage of peripheral CD4^+^CD25^+^ T cells in vitiligo patients and disease activity according to VIDA score. This result is in agreement with previous studies that detected reduced percentage of CD4^+^CD25^+^ T cells in peripheral blood of progressive vitiligo patients compared to the patients with stable vitiligo, and functional analysis of peripheral Tregs in vitiligo patients showed a correlation of Tregs functions with the disease status [18, 20]. Moreover, Lili et al. observed that the percentage of circulating Treg cells increased significantly after treatment-induced disease stabilization [19].

In the present study the percentage of peripheral FoxP3^+^ cells in vitiligo patients was also significantly low compared to healthy controls and this matches previous reports [18, 23]. On the other hand, Ben Ahmed et al. suggested a recruitment of Tregs from the peripheral blood to the site of vitiligo which was further corroborated by the significant increase of FoxP3 expression in the vitiliginous skin of patients [20].

Among the clinical parameters of our vitiligo patients, a significant negative correlation was detected between the percentage of FoxP3^+^ cells in peripheral blood and VIDA score. This comes in agreement with a previous study in which VASI, VIDA, and stress scores correlated negatively with FoxP3^+^ cell levels both in peripheral blood (by real time PCR) and in skin samples (by immunohistochemistry) [23]. However, no evidence of a correlation between Tregs and either activity or duration of vitiligo was found by Moftah et al. in 2014 [24].

In the current study, a significant positive correlation was observed between the peripheral percentage of FoxP3^+^ cells and that of CD4^+^CD25^+^ T cells in vitiligo patients. This matches the results of previous studies [18, 25]. Yagi et al. showed in their study that FoxP3^+^ is preferentially and stably expressed in Tregs cells in humans and that ex vivo retroviral gene transfer of FoxP3^+^ can convert human naive CD4^+^ T cells into a regulatory T-cell phenotype similar to CD4^+^CD25^+^ Tregs cells, indicating that FoxP3^+^ may be a master regulatory gene for the function of CD4^+^CD25^+^ Treg cells in humans [26].

In vitiligo, the dysfunction of Tregs could result from the alteration of generation, peripheral survival, activation, suppressor mechanisms, or migratory behavior of these cells [27, 28]. Decreased percentage of peripheral CD4^+^CD25^+^ Tregs and decreased expression of FoxP3^+^ might impair the suppressive activity of Treg cells on cell proliferation with breakage of tolerance to melanocyte self-antigens which could contribute to the pathogenesis of vitiligo [17]. A previous study had indicated that an imbalance of CD8^+^ cytotoxic T lymphocytes and natural Tregs in frequency and function might be involved in the progression of generalized vitiligo. The deficiency and dysfunction of natural Treg cell subpopulations were thought to help in a global expansion and widespread activation of CD8^+^ T-cell population, which could result in the destruction of melanocytes and an elevated frequency of associated autoimmune diseases in generalized vitiligo patients [19].

Regulatory cytokines produced by Treg cells, such as IL-10 and TGF-*β*, are suggested to be related to the stability of vitiligo [29]. In 2013, Tembhre et al. detected increased serum levels of IL-10, IL-13, and IL-17A and decreased concentrations of TGF-*β*1 in patients with vitiligo and that might facilitate the melanocyte cytotoxicity; meanwhile treatment with NB-UVB was capable of elevating TGF-*β* levels, suggesting that Treg cytokines might play an important role in repigmentation [30]. Hegazy et al. proposed that restoration of the balance between Th17 and Tregs might represent a novel pathway for the improvement that NB-UVB exerts in vitiligo patients [31]. In the same regard, Eby et al. hypothesized that promoting Treg skin homing through enhanced expression of CCL22 might suppress depigmentation in vitiligo [32]. There is a future potential to utilize Treg and Treg-friendly therapies to replace current general immunosuppressives and induce tolerance as a path towards a drug-free strategy without associated toxicities in autoimmune diseases including vitiligo [33].

In conclusion, our results suggest that peripheral Treg depletion with impaired immune downregulatory function might participate in the autoimmune conditions beyond the pathophysiology and activity of nonsegmental vitiligo. Effective Treg cell-based immunotherapies might be a future hope for patients with progressive vitiligo. Larger scale studies might provide a better understanding of Tregs populations and functions, and that might help in successful treatment of autoimmune responses in active vitiligo based on induction or expansion of antigen-specific Tregs.

## Figures and Tables

**Table 1:** Demographic and clinical characteristics of study participants.

	Vitiligo patients	Control group
	*n* = 80	*n* = 60
Range of age in years, mean (SD)	9–60, 27.25 (14.32)	20–50, 33.9 (9.5)
Gender, *n* (%)		
Male	36 (45)	36 (60)
Female	44 (55)	24 (40)
Range of disease duration in years, mean (SD)	0.33–49, 5.34 (4.81)	NA
Type of vitiligo, *n* (%)		
Localised	36 (45)	NA
Generalised	32 (40)	
Acrofacial	12 (15)	
Range of VASI score, mean (SD)	10–90, 42.3 (26.7)	NA
VIDA score, *n* (%)		
0	16 (20)	NA
+1	12 (15)	
+2	32 (40)	
+3	16 (20)	
+4	4 (5)	

NA: not applicable. SD: standard deviation.
